# Daily changes in sleep stages and associated cardiovascular parameters during pregnancy: using a wearable device

**DOI:** 10.3389/fgwh.2025.1622895

**Published:** 2026-02-10

**Authors:** Yuqing Guo, Qi Xu, Laura Narvaez, Nikil Dutt, Priscilla Kehoe, Annie Qu

**Affiliations:** 1 Sue and Bill Gross School of Nursing, University of California, Irvine, CA, United States; 2 Department of Statistics & Data Science, Carnegie Mellon University, Pittsburgh, PA, United States; 3 Department of Computer Science, University of California, Irvine, CA, United States; 4 Department of Statistics and Applied Probability, University of California Santa Barbara, Santa Barbara, CA, United States

**Keywords:** digital health technologies, wearable device, remote patient monitoring, sleep stage, autonomic nervous system, cardiovascular physiological phenomena, pregnancy

## Abstract

**Objective:**

Growing evidence links sleep disturbances with gestational hypertension and/or preeclampsia. Most studies rely on self-reported surveys that cannot capture sleep stages as objective indicators of cardiovascular health. The objective of this study was to utilize a wearable device to describe changes in nightly sleep stages during pregnancy and investigate their relationships with resting heart rate (HR) and heart rate variability (HRV).

**Methods:**

This longitudinal descriptive study encompassed 981 observation days among 14 pregnant women, 86% of whom self-identified as Hispanic. The Oura ring obtained nighttime sleep and cardiovascular data every 5 min, including deep, Rapid Eye Movement (REM), light sleep, and awake, as well as HR and HRV. The frequency and duration of each sleep stage occurrence indicated daily sleep characteristics. Linear mixed models were employed to detect daily changes in sleep stages and to investigate their associations with cardiovascular parameters while adjusting for maternal age, and pre-pregnancy body mass index.

**Results:**

Significant daily changes occurred in deep/light sleep and awake (all *p* values < 0.05), but not in REM. The deep sleep onset significantly increased (*p* = 0.03), while the deep sleep occurrence frequency decreased (*p* < 0.001). The duration per light sleep decreased (*p* = 0.04) while awake increased (*p* = 0.009). More deep sleep was significantly associated with lower HR but higher HRV (all *p values* < 0.01). REM sleep had opposite patterns, associated with increased HR and decreased HRV (all *p values* < 0.01). Light sleep/awake showed no significant associations with HR/HRV.

**Discussion:**

This is the first study using a wearable device to describe daily associations between sleep and cardiovascular parameters during pregnancy among primarily Hispanic pregnant women. The findings suggest that non-invasive wearable devices monitoring deep sleep and parasympathetic nervous system activity could inform digital interventions on how to enhance deep sleep and promote cardiovascular health in pregnant women.

**Conclusion:**

Wearable device monitoring can identify critical changes in sleep during pregnancy, particularly the relationship whereby greater deep sleep is associated with favorable cardiovascular health markers. These findings lay the groundwork for developing personalized, technology-enabled maternal health interventions to promote cardiovascular wellness during pregnancy.

## 1 Introduction

Disturbed sleep is a common complaint among pregnant women (1, 2). Most of these studies have been assessed with questionnaires such as the Pittsburgh Sleep Quality Index: PSQI (1–3). The self-reported surveys could not capture certain sleep stages that reflect critical aspects and essential indicators of physical and psychological well-being and health (4). According to the American Academy of Sleep Medicine, sleep includes four stages: deep, Rapid eye movement (REM), light, and wake (5). Specifically, deep sleep plays an essential role in restorative effects in terms of promoting the immune system, repairing muscles, bones, and tissues, regulating glucose metabolism, and replenishing energy (6, 7). REM sleep is characterized by dreaming, emotional processing, memory consolidation, cognitive development, and preparation for wakefulness (8, 9). Light sleep includes the transitional phase from wake to sleep and the intermediate phase of sleep, during which the entire body enters a more subdued state (6). During wake, an individual is typically alert and responsive (5). Emerging evidence shows the feasibility of using wearable technology to monitor objective sleep patterns in the home setting (10–12).

Investigation of the longitudinal pattern of objective sleep stages in pregnant women is relatively sparse (4). While a few studies measured objective sleep data using polysomnography (PSG) in a lab setting for one day in one or two trimesters (13), our previous research employed a wearable device (Oura ring) to collect objective sleep parameters at home during pregnancy (14). Its results revealed a decrease in weekly deep sleep without significant changes in REM sleep, and importantly, identified maternal age and pre-pregnancy body mass index (BMI) as moderators. Women ≥ 30 years old experienced a more substantial reduction of deep sleep, while those (women ≥ 30) with a pre-pregnancy BMI ≥25 showed increased REM sleep duration (14). Notably, these studies have primarily focused on describing sleep changes (e.g., decreased total sleep time) over pregnancy, leaving a gap in understanding the possible daily patterns that influence these sleep changes. Our present study addressed this gap by utilizing a wearable device (Oura ring) to obtain sleep parameters every 5 min (rather than weekly aggregated data), enabling a more detailed description of daily changing patterns in characteristics of each sleep stage during pregnancy.

The American Heart Association postulates that sleep is now recognized as an essential component of cardiovascular health (15). During deep sleep, heart rate (HR) tends to drop, while in REM sleep, HR tends to increase (16). Reduction in deep sleep increases the risk for blunted dipping nocturnal blood pressure and the development of hypertension in healthy non-pregnant adults (17, 18). As for pregnant women, there is a limited understanding of the relationships between sleep and cardiovascular parameters during pregnancy. Accumulating evidence has demonstrated that sleep-disordered breathing (e.g., obstructive sleep apnea) is an independent risk factor for gestational hypertension and preeclampsia (19–22). Additional studies show the relationships between other types of self-reported sleep disturbances (e.g., short or long sleep duration, poor sleep quality) and elevated blood pressure, gestational hypertension and/or preeclampsia (23, 24). Furthermore, HR and heart rate variability (HRV) have been identified as non-invasive clinical markers for cardiovascular health (25, 26). Elevated resting HR and/or decreased HRV are independent risk factors for cardiovascular disease, such as stroke and myocardial infarction (25, 26). However, there is a dearth of evidence describing objective sleep and associated cardiovascular parameters in pregnant women. Thus, the objective of this study was to examine the longitudinal daily sleep stages and their relationships with cardiovascular parameters during pregnancy using a wearable device (Oura ring) that provides data every 5 min.

## 2 Materials and methods

### 2.1 Study design and sampling

We used a longitudinal, prospective, observational design to understand the feasibility of using wearable technology to examine biopsychosocial changes in underserved pregnant women. The University Office of Research Institutional Review Board (IRB) approved the use of verbal informed consent for the study. The trained research coordinator explained the study to all participants using an IRB-approved Study Information Sheet. During the recruitment process, the participants' consent for participation in the research and publication of results was obtained before the study commenced. The consenting information was recorded in the research protocol. The required ethical standards were met for all research procedures.

Convenience sampling was used to recruit participants. As we began the recruitment process, the COVID-19 Stay-At-Home Restriction was mandated, which influenced participant interactions and required virtual contact only. The study flyer was shared with community partners working with underserved perinatal women in Orange County, California. Thus, most of the women recruited were Hispanic. The inclusion criteria were pregnant women aged 18–40, with a healthy singleton pregnancy without complications at enrollment, and access to a smartphone. The research coordinator screened all potential participants and informed them about the research, including its possible benefits and risks. With verbal consent, as approved by the IRB, participants were enrolled in the study. All the participants received an IRB-approved Study Information Sheet, ensuring they could contact the research team and/or IRB with any inquiries. The women were instructed to wear the Oura ring as much as possible throughout their pregnancy, particularly at night. The detailed study procedure was published in a prior study (14).

Fifty-three potential women were screened, and twenty were eligible to participate and consent. Of 20 participants, two dropped out early due to family circumstances, and four had insufficient 5-minute data. Thus, 981 observation days from 14 women were included in this current study. All women were enrolled at ≥10 gestational weeks (mean = 17.93 ± 5.76). Of 14 participants, 12 (86%) self-identified as Hispanic with an average age of 28.57 years (*SD* = 4.18). More than half of the women (8, 57%) were overweight or obese. Five (36%) had some college education or an associate degree, and 9 (64%) had a bachelor's or master's degree. Five (36%) were first-time mothers. Four (29%) had public health insurance. Thirteen (93%) were married and lived with the father of the baby. Approximately one-third (*n* = 4, 29%) had a history of depression/anxiety. Two participants experienced COVID-19 while participating in the study, and one participant developed gestational diabetes and hypertension during the current pregnancy.

### 2.2 Data collection procedure

Research protocols adhered to the COVID-19 Stay-At-Home Restrictions. REDCap, a secure data collection platform, was used to collect demographics. The Oura ring is a waterproof multi-sensor wearable device that detects physiological signals using an optical pulse waveform from the participant's finger. The data are transferred automatically via Bluetooth to an App installed on the participants' smartphones (Oulu, Finland). Each woman was given a ring size that seemed appropriate for comfort, and then the ring was shipped to her with a set of standardized instructions on installing and using the ring. In the current study, pregnant women were instructed to wear the Oura ring for 24 h, particularly every night during pregnancy. The ring, synchronized with the participant's mobile app, displayed a summary of sleep parameters. During the study, the research team and participants communicated virtually. The study occurred from October 2020 to December 2021. Each participant received a $200 gift card as compensation for their participation in the study.

### 2.3 Measures

Self-reported demographic data, such as maternal age, education, ethnicity, parity (the number of pregnancies carried to at least 20 weeks), and pre-pregnancy BMI, were gathered through REDCap. This Oura ring (Generation 2) measured nocturnal sleep at home (napping was not assessed). Nocturnal sleep parameters are detected and measured using a combination of nighttime movement, resting HR, and HRV, as well as pulse wave variability amplitude collected from photoplethysmography, a negative temperature coefficient thermistor, and a 3-D accelerometer, employing machine learning methodology (27). Sleep stages comprise the hours in deep, REM, and light sleep as well as awake (i.e., wakefulness occurring after sleep onset) (28). The Oura ring was also utilized to measure cardiovascular parameters, including resting HR and HRV. HRV is indicated by the root mean square of successive differences (RMSSD, in milliseconds) of inter-beat intervals (29). The accuracy of the Oura ring has been validated through studies comparing its performance to the gold standard tools, such as PSG and/or Electrocardiography (27, 30, 31).

The Oura ring collects sleep and cardiovascular data every 30 s throughout the night. We obtained permission from Oura to utilize the 5-minute data for each parameter. These 5-minute data were used to measure the onsets of deep and REM sleep, as well as the daily characteristics of each sleep stage, including the frequency and duration of each occurrence. The frequency of a sleep stage refers to the number of times each stage occurs (i.e., how often does each sleep stage occur per night?). The duration refers to the average length of each sleep stage per occurrence (i.e., how long does each sleep stage last per night?). Furthermore, 5-minute HR and HRV were also used to assess two crucial daily cardiovascular parameters.

### 2.4 Statistical analysis

All pre-processing and statistical analyses were performed in R (version 4.2.0 for Mac), with *p* ≤ 0.05 indicating statistical significance. There were five steps. First was to examine the daily changes in sleep parameters throughout pregnancy. We developed eight separate linear mixed models—each corresponding to one of the following response variables indicating daily changes in sleep stages: frequency and duration of deep, REM, light sleep, and awake. In each model, the response variable was measured over multiple days during pregnancy, and day of pregnancy (i.e., gestational day) was incorporated as both a fixed and a random effect to account for within-subject correlations. The fixed effect estimates and associated *p*-values for these models are presented in Table 1. Second, we assessed the associations between daily sleep parameters and HR using a linear mixed model that similarly accounted for longitudinal correlations within subjects. In the unadjusted model (Model 1), HR served as the response variable, with the eight sleep parameters and day of pregnancy included as fixed and random effects. We further adjusted for potential confounders in Model 2, controlling for maternal age and pre-pregnancy BMI. The results of these analyses are summarized in Table 2. Third, we employed an analogous modeling approach to investigate the relationship between sleep parameters and HRV. Model 1 was unadjusted, while Model 2 was adjusted for maternal age and pre-pregnancy BMI. The corresponding findings are presented in Table 3. For the analyses presented in Table 2 and 3, we applied a Bonferroni correction to account for the eight sleep parameter comparisons and minimize the risk of false discoveries, using an adjusted significance threshold of 0.05/8 = 0.00625. Fourth, we conducted sensitivity analyses to assess the robustness of the findings by including only women with participation rates of 70% or higher. Participation rate was calculated as the number of actual Oura ring observations divided by total study days (from the first to the last day of Oura ring observation). The results remained consistent with similar coefficients. Fifth, we performed subgroup analyses to confirm our findings by stratifying participants based on age (<30 or ≥30 years) and pre-pregnancy BMI (< 25 or ≥25 kg/m^2^) separately.

**Table 1:** Longitudinal patterns of sleep stages during pregnancy.

Sleep stage	Variable	Daily average change (SD)	*p*-value
Deep	Frequency^a^	−0.008 (0.002)	**<0**.**0001**
	Duration^b^ (min)	−0.013 (0.009)	0.14
REM	Frequency^a^	−0.003 (0.002)	0.17
	Duration^b^ (min)	0.007 (0.009)	0.43
Light	Frequency^a^	−0.002 (0.003)	0.58
	Duration^b^ (min)	−0.009 (0.005)	**0**.**04**
Awake	Frequency^a^	0.007 (0.004)	0.10
	Duration^b^ (min)	0.020 (0.008)	**0**.**009**

a Frequency: Frequency of each sleep stage occurrence per day.

b Duration: Average duration of each sleep stage occurrence per day.

Note: **bold** ***p*****-values**: *p*-values reach statistical significance of *p* ≤ 0.05.

**Table 2:** Daily sleep stages associated with heart rate.

Variable	Model 1 (unadjusted model)		Model 2 (adjusted model)	
	Coefficient (SD)	*P*-value	Coefficient (SD)	*P*-value
Deep Frequency^a^	−0.549 (0.090)	**<0** **.** **0001**	−0.528 (0.077)	**<0**.**0001**
Deep Duration^b^	−0.078 (0.021)	**0**.**0002**	−0.075 (0.019)	**0**.**0001**
Light Frequency^a^	−0.018 (0.066)	0.7868	−0.031 (0.066)	0.6332
Light duration^b^	−0.032 (0.032)	0.3254	−0.033 (0.027)	0.2158
REM Frequency^a^	0.344 (0.105)	**0**.**0011**	0.339 (0.100)	**0**.**0007**
REM duration^a^	0.917 (0.032)	**0**.**0044**	0.095 (0.031)	**0**.**0025**
Awake Frequency^a^	−0.062 (0.0644)	0.3400	−0.051 (0.062)	0.4082
Awake Duration^b^	0.013 (0.019)	0.5130	0.015 (0.020)	0.4360
Day of Pregnancy	0.045 (0.015)	**0**.**0024**	0.046 (0.014)	**0**.**0014**
Maternal age	–	–	0.420 (0.433)	0.3533
Pre-pregnancy BMI	–	–	1.561 (0.532)	**0**.**0136**

a Frequency: frequency of each sleep stage occurrence per day.

b Duration: average duration of each sleep stage occurrence per day.

Note: **bold** ***p*****-values**: *p*-values reach statistical significance of *p* ≤ 0.05.

**Table 3:** Daily sleep stages associated with heart rate variability.

Variable	Model 1 (unadjusted model)		Model 2 (adjusted model)	
	Coefficient (*β*)	*P*-value	Coefficient (*β*)	*P*-value
Deep frequency^a^	1.566 (0.227)	**<0**.**0001**	1.559 (0.239)	**<0**.**0001**
Deep duration^b^	0.150 (0.044)	**0**.**0007**	0.153 (0.046)	**0**.**0009**
Light frequency^a^	−0.108 (0.140)	0.4398	−0.095 (0.139)	0.4960
Light duration^b^	0.033 (0.081)	0.6862	0.042 (0.076)	0.5813
REM frequency^a^	−1.014 (0.340)	**0**.**0029**	−1.044 (0.337)	**0**.**0020**
REM duration^b^	−0.296 (0.079)	**0**.**0002**	−0.300 (0.080)	**0**.**0002**
Awake frequency^a^	0.279 (0.167)	0.0943	0.292 (0.172)	0.089
Awake duration^b^	0.095 (0.060)	0.1129	0.086 (0.059)	0.1468
Day of pregnancy	−0.030 (0.018)	0.0918	−0.035 (0.018)	0.0569
Maternal age	–	–	−1.029 (0.391)	**0**.**0234**
Pre-pregnancy BMI	–	–	−0.132 (0.478)	0.7867

a Frequency: frequency of each sleep stage occurrence per day.

b Duration: average duration of each sleep stage occurrence per day.

Note: **bold** ***p*****-values**: *p*-values reach statistical significance of *p* ≤ 0.05.

## 3 Results

### 3.1 Longitudinal changes in sleep stages during pregnancy

**Figure 1:**
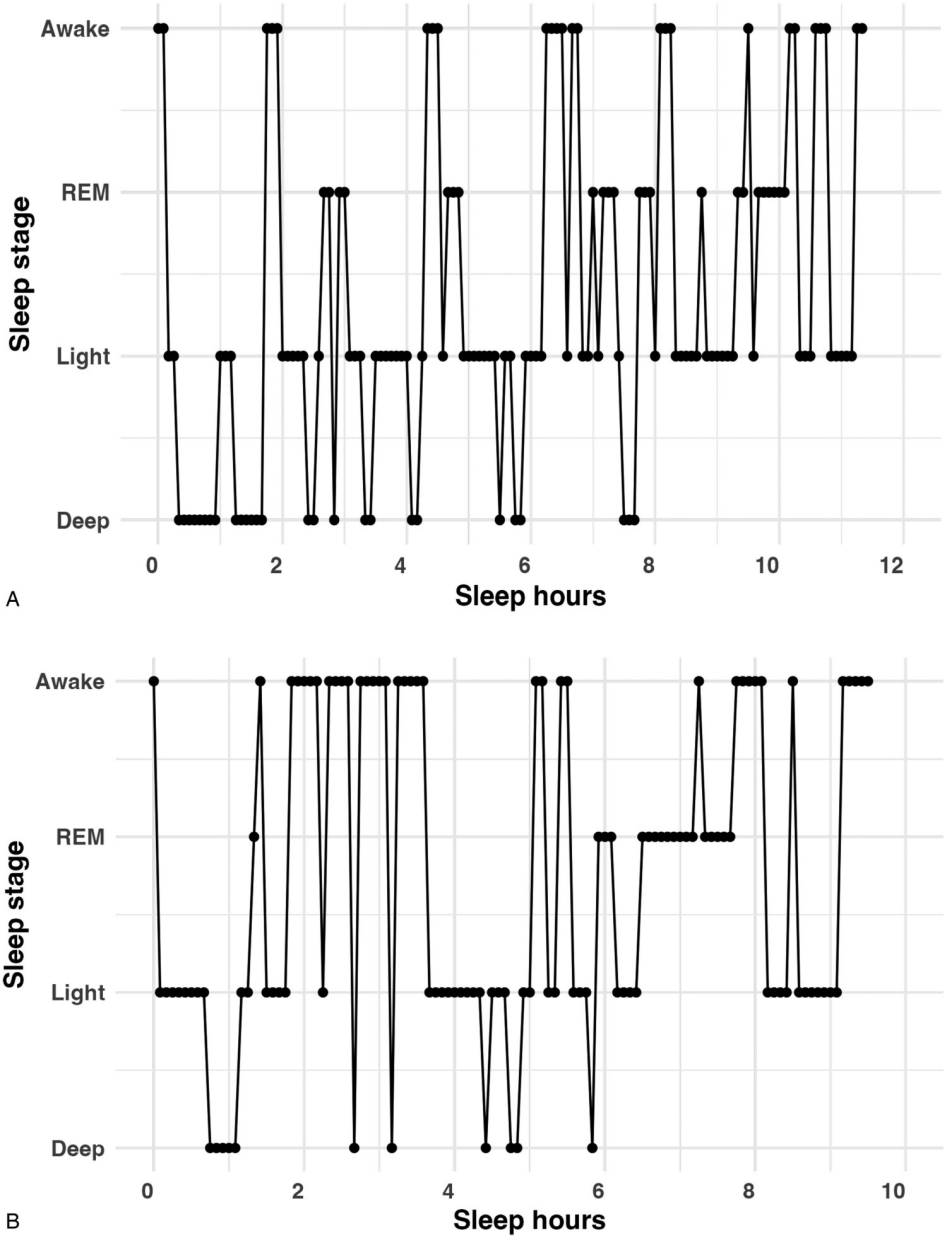
**(A)** Sleep cycles in gestational week 15. **(B)** Sleep cycles in gestational week 38 (the same participant). Each dot/mark represents a 5-minute sleep stage occurrence.

Significant daily changes occurred in deep and light sleep as well as awake, as shown in Table 1. Specifically, during pregnancy, we found that the daily onset of deep sleep increased significantly (0.07 ± 0.03 min/day, *p* = 0.03), while the frequency of deep sleep (i.e., the number of times it occurred each night) decreased daily by 0.008 ± 0.002 min/day (*p* < 0.001). Regarding REM sleep, there were no significant daily changes in onset, frequency, or duration. Light sleep and awake showed opposite patterns in daily duration (all *p values* < 0.05). Figure 1A and 1B demonstrate two sleep histograms representing one individual's change in sleep stages during pregnancy. Each dot/mark in these figures refers to a 5-minute sleep stage occurrence. Notably, from gestational week 15–38, deep sleep started later, occurred less frequently, and had a shorter duration for each occurrence.

### 3.2 Associations between daily sleep and cardiovascular parameters

We found that only deep and REM sleep (but not light or awake) were significantly associated with resting HR and HRV. To control for multiple comparisons and avoid an inflated false discovery rate, we applied a Bonferroni correction using a significance threshold of 0.05/8 = 0.00625. All statistically significant sleep parameters based on raw *p*-values remained significant after this correction in Tables 2 and 3.

During pregnancy, there were negative associations between deep sleep and HR as opposed to positive associations between REM sleep and HR (see Table 2). Those associations remained significant after controlling for additional covariates, including maternal age and pre-pregnancy BMI. Specifically, every additional deep sleep occurrence decreased HR by 0.528 bpm, and each additional minute of deep sleep occurrence reduced HR by 0.075 bpm. Conversely, every additional REM sleep occurrence increased HR by 0.339 bpm, and each additional minute of the length of REM sleep occurrence increased HR by 0.095 bpm. Moreover, Table 3 shows that deep sleep was positively associated with HRV, while REM sleep was negatively associated with HRV. Specifically, every additional deep sleep occurrence increased HRV by 1.559 milliseconds (ms), and each additional minute of the length of the deep sleep occurrence increased HRV by 0.153 ms. In contrast, every additional REM sleep occurrence decreased HRV by 1.044 ms, and each additional minute of the length of REM sleep occurrence decreased HRV by 0.30 ms. Furthermore, HR significantly increased across gestational days, and greater pre-pregnancy BMI was associated with an even higher HR (see Table 2). Additionally, greater maternal age was also significantly associated with lower HRV (see Table 3). Notably, the subgroup analyses confirmed that the associations between deep/REM sleep and HR/HRV were preserved across age <30 vs. ≥30 or pre-pregnancy BMI (< 25 vs. ≥25). The magnitude of coefficients and statistical significance varied slightly depending on the specific deep/REM sleep metric examined: occurrence frequency vs. duration of each occurrence. However, the small sample sizes in these subgroup analyses substantially limited statistical power to detect consistently significant effects.

## 4 Discussion

### 4.1 Continuous changes in sleep characteristics during pregnancy

Our study found that as pregnancy progressed, it took longer for women to enter their first deep sleep stage, and the number of deep sleep occurrences decreased. These findings confirmed previous studies using the Oura ring or PSG, which showed decreased deep sleep duration during pregnancy (13, 14). We extended these studies by identifying increased latency to the first deep sleep and reduced number of deep sleep occurrences as potential mechanisms underlying the shortened deep sleep duration. Importantly, we provided new evidence on the longitudinal change in deep sleep onset among pregnant women. The changing patterns of deep sleep parameters observed in our study could have clinical implications. Prior studies have shown that decreased deep sleep is associated with increased pro-inflammatory responses, such as elevated C-reactive protein and TNF-alpha (32, 33). Higher levels of pro-inflammatory markers are known risk factors for adverse pregnancy outcomes (e.g., gestational diabetes, preeclampsia, recurrent spontaneous pregnancy loss (32, 34–36). Therefore, investigating how various characteristics of deep sleep (onset, frequency, and/or duration per deep occurrence) could predict perinatal outcomes is essential. This research could provide evidence to support the development of early interventions aimed at reducing adverse consequences among pregnant and postpartum women.

Interestingly, we found that the characteristics of REM sleep remained similar during pregnancy in terms of onset, frequency, and duration per REM occurrence. Our findings support one PSG study that showed no significant change in REM sleep duration and onset in pregnant women without complications (37). However, another study found a reduction in REM using PSG in similar populations of pregnant women (13). Additional studies are needed to investigate REM sleep patterns in pregnant women with medical conditions. For example, increased REM onset with PSG was found in preeclamptic patients who were on clonidine to control their hypertension compared to healthy pregnant women, and a much longer latency of first REM onset was attributed to medication response (38). Our study provides preliminary evidence of the potential use of a wearable device to continuously monitor the responses of preeclamptic patients before and after prescribed medications.

### 4.2 Associations between sleep and cardiovascular parameters

To our knowledge, it appears that our study is the first to report that during pregnancy, greater deep sleep (frequency and/or duration) was associated with lower resting HR and higher HRV, whereas greater REM sleep (frequency and/or duration) was associated with higher HR and lower HRV. Our results are consistent with previous findings, which showed similar relationships between deep sleep/REM and HR/HRV in non-pregnant populations (39–41). In other words, our results support the dynamic relationship between sleep stages and the autonomic nervous system (ANS) in that deep sleep is associated with parasympathetic modulation, whereas REM sleep is related to sympathetic regulation (40, 41). The contribution of this study is to add evidence of longitudinal relationships between sleep and cardiovascular parameters among pregnant women in the home setting. Specifically, we extended the existing literature by highlighting that daily changes in deep and REM sleep could be sensitive indicators of cardiovascular health. Interestingly, despite increased wakefulness being one of the complaints in pregnant women (1–3), this study found no significant associations between awake and HR/HRV. Noteworthy is that subgroup results supported relationships between deep/REM sleep and HR/HRV, though slight variations emerged depending on the specific sleep characteristic (frequency or duration). Future research is necessary to confirm these findings with a large sample size and better understand these relationships among pregnant women with pre-pregnancy health conditions and/or pregnancy complications. In addition to sleep stages, we also found that other factors influence cardiovascular health. Our study found positive associations between pre-pregnancy BMI and HR as well as negative associations between maternal age and HRV. These results support existing literature by confirming that pre-pregnancy BMI is a key factor impacting resting HR (42). Additionally, our findings are aligned with prior research showing that HRV collected during sleep is influenced by maternal age (43).

Our results have critical clinical implications. We revealed evidence of significant associations between sleep stages and the ANS in pregnant women, similar to findings in healthy nonpregnant populations. For example, as we and others have found, deep sleep is associated with decreased sympathetic activity, which is linked to reduced resting HR and blood pressure (17, 18, 44). Research using PSG has demonstrated that shorter deep sleep duration predicts higher rates of hypertension (18). Additionally, no-dipping (decreased) or reverse-dipping (increased) HR during sleep is an independent risk factor for hypertension (45). In pregnant populations, women with hypertensive disorders demonstrated lower HRV compared to healthy pregnant women in laboratory settings (46). A recent systematic review found reduced HRV in pregnant women with preeclampsia compared to healthy counterparts, indicating dysregulated ANS with sympathetic hyperactivity and parasympathetic hypoactivity (47). Additionally, approximately 78% of pregnant women with hypertensive disorders of pregnancy and obstructive sleep apnea exhibited non-dipping blood pressure patterns (48). These findings highlight the importance of investigating the dynamic relationship between sleep stages and ANS function in predicting cardiovascular health among pregnant women (49).

In summary, our study demonstrated that daily sleep stage characteristics (frequency and/or duration) were associated with cardiovascular parameters using digital technology monitoring. Notably, we detected decreased frequency of deep sleep episodes each night during pregnancy and found its association with the cardiovascular system. These results imply that daily variations in these sleep characteristics may serve as potential predictors of perinatal outcomes. Our study suggests that wearable technology, such as the Oura ring, could enable continuous at-home monitoring of targeted biomarkers for perinatal women (11). This approach opens new avenues for personalized, technology-driven perinatal health promotion. Future studies could potentially develop interventions that enhance deep sleep to promote cardiovascular health in pregnant women.

### 4.3 Limitations

Despite the novel findings of this study, several limitations should be acknowledged. First, since the purpose of the parent study was to pilot the acceptability of using the Oura ring to understand objective sleep parameters among healthy pregnant women, no power analysis was conducted. The small sample size limits further examination of how other socioeconomic and clinical factors (e.g., marital status, parity, pregnancy history) influence sleep and cardiovascular patterns. Second, data on sleep breathing disorders were not collected and therefore not included as a covariate. Given that overweight or obese women have an increased risk of developing sleep apnea, the interpretation of the results requires caution. Third, wearables may influence participant behavior since users could access their sleep and cardiovascular data directly from their smartphones, potentially introducing bias. Fourth, cardiovascular health assessment was restricted to resting HR and HRV measurements, representing only partial indicators of cardiovascular function. Fifth, emerging evidence indicates that the relationship between sleep and HR/HRV is bidirectional (50, 51). While we identified significant associations between sleep stages and HR/HRV, the observational nature of this study precludes any causal inferences. Future studies should investigate how sleep parameters influence cardiovascular outcomes, particularly in at-risk pregnant women. Finally, generalizability is limited because most participants were Hispanic pregnant women, with nearly half being overweight or obese.

Future studies should extend this research by using larger, more diverse samples that include various racial/ethnic groups and women with different BMI profiles, and by incorporating a broader range of potential confounding variables (e.g., sleep-disordered breathing) and cardiovascular assessments (e.g., blood pressure, cardiac output, vascular resistance). Additionally, longitudinal designs are needed to investigate how sleep parameters causally influence cardiovascular outcomes and to provide a more comprehensive understanding of cardiovascular health during pregnancy, particularly among at-risk pregnant women.

## 5 Conclusions

Our study contributes to the existing literature by quantifying daily changes in objective sleep stages and identifying potential factors for these changes among primarily Hispanic pregnant women in U.S. home settings. Using a digital wearable device, we detected longitudinal objective sleep changes during pregnancy, primarily characterized by reduced deep sleep, including delayed onset of the first deep sleep and fewer deep sleep occurrences. Furthermore, our results indicate that longer deep sleep and shorter REM sleep are associated with the autonomic nervous system, characterized by lower resting HR and higher HRV. These findings provide opportunities for future interventions that could potentially promote deep sleep, monitored through non-invasive and convenient wearable devices, to improve cardiovascular health in pregnant women.

## Data Availability

The raw data supporting the conclusions of this article will be made available by the authors, without undue reservation.
